# Healthcare Supported by Data Mule Networks in Remote Communities of the Amazon Region

**DOI:** 10.1155/2014/730760

**Published:** 2014-10-29

**Authors:** Mauro Margalho Coutinho, Alon Efrat, Thienne Johnson, Andrea Richa, Mengxue Liu

**Affiliations:** ^1^Centro de Ciências Exatas e Tecnologia, Universidade da Amazônia, 66060-902 Belém, PA, Brazil; ^2^Department of Computer Science, University of Arizona, Tucson, AZ 85721-0077, USA; ^3^Computer Science and Engineering, CIDSE, Arizona State University, Tempe, AZ 85287-8809, USA

## Abstract

This paper investigates the feasibility of using boats as data mule nodes, carrying medical ultrasound videos from remote and isolated communities in the Amazon region in Brazil, to the main city of that area. The videos will be used by physicians to perform remote analysis and follow-up routine of prenatal examinations of pregnant women. Two open source simulators (the ONE and NS-2) were used to evaluate the results obtained utilizing a CoDPON (continuous displacement plan oriented network). The simulations took into account the connection times between the network nodes (boats) and the number of nodes on each boat route.

## 1. Introduction

Data mule is an acronym for (mobile ubiquitous LAN extension). It is related to vehicles that physically carry a computer with a storage device and a limited telecommunication module (usually Wi-Fi) between remote areas in order to effectively create a data communication link [1]. A* delay-tolerant network (DTN)* is an architecture that provides a common method for interconnecting heterogeneous gateways or proxies that employ store-and-forward message routing to overcome communication disruptions [2]. Both technologies are tolerant to disconnections in the network and are often complementary. However, despite the fact that these technologies do not adhere to the principle of ubiquitous computing, they open the doors to hundreds of applications whose integration was not possible earlier, mostly due to the high costs, or even infeasibility, of implementing a networked infrastructure in some scenarios.

One of these scenarios is the Marajó Archipelago, located in the Amazon region of Brazil, which occupies a large fluvial area of 104,142 Km^2^ (approximately 40,700 sq mi) [3]. The largest island on the archipelago, the Marajo Island, has roughly the same size as Switzerland and is the largest island in the world to be surrounded by freshwater. According to the last Brazilian census [4], only 43% of the archipelago's population of 487,010 inhabitants lives in urban areas. There are no bridges linking the islands to the mainland, and boats are by far the main means of transportation in the region. Some cities, like Afua (known as the “Venice of the Amazon”), are completely built on water. In these places, socioinfrastructure problems abound, especially health related problems, as there are few physicians available in the remote communities. Most medical care is done through sporadic government programs that involve the displacement of a team to serve the population, in particular in the outlying areas. The goal of this paper is to present an alternative telemedicine infrastructure, focused on performing remote triage and prioritization of medical care in these remote regions of the Amazon rain forest. We implement a telemedicine infrastructure over a CoDPON (continuous displacement plan oriented network) data mule system [5], commonly applied in scenarios where nodes have predefined routes. We present a preliminary evaluation of the capabilities of our proposed telemedicine infrastructure by means of simulations. We simulate the implementation of a remote ultrasound video analysis service for pregnant women in the Marajó Archipelago, as part of their prenatal treatment, over our data mule network.

This paper is organized as follows: Section 2 presents related work, Section 3 presents the system architecture, Section 4 presents a description of the remote ultrasound telemedicine application, and Section 5 presents our simulation studies.  Section 6 concludes this work and presents suggestions for the improvement of services through the use of fountain codes.

## 2. Related Work

### 2.1. DTN and Data Mule

The first terrestrial DTN applications were proposed for sensor networks [6–8]. Soon other applications emerged for integration of remote communities like Daknet [9] that provided low-cost digital communication, allowing remote villages to leap from the expense of traditional connectivity solutions and the deployment of a full-coverage broadband wireless infrastructure. Another proposal, named MotoPost, was presented in [10]. The concept was developed to address the communication and information access needs of remote rural villages in India that do not have access to communication technologies.

The epidemic routing is a routing protocol for DTN. This algorithm is named this way because messages are spread like infectious diseases [11]: when two nodes come into contact, the first node sends the second node a summary vector containing information that uniquely identifies the bundles of information (messages) it has. The second node then sends the first node any bundles that it has and the first does not have. This takes place in both directions whenever two nodes come into contact [12]. For a moderate volume of data, epidemic routing is considered as one of the most effective routing schemes for delay-tolerant networks; indeed, epidemic routing allows for the shortest routing delays [13].

These and other efforts sensitized the scientific community to produce new tools to evaluate the impact of DTNs in different scenarios. One of the most popular DTN simulators “the ONE” [8]—opportunistic network environment—has rich features available for simulating DTNs with numerous mobility models. It is a useful tool for platform for implementation and evaluations of new ideas. Recently, Khalid et al. [14] evaluated the concept of checkpoint (CP). CPs are autonomous high-end wireless devices with a large buffer for storage and are responsible for temporarily storing messages to be forwarded. According to the authors, the basic components of a CP node are processor, memory, solar powered batteries, multiple interfaces (standard Ethernet; 802.11b/g/n; Bluetooth), GPS, and storage. The results of simulation in an area of 4 × 3 KM (the size of the city of Fargo, ND, USA) show that message delivery ratio improves by increasing the number of checkpoints in the area.

### 2.2. “Store-and-Forward” Telemedicine

In scenarios where real-time communication network might not be a practical solution for the delivery of large files, “store-and-forward” approaches could come in handy. Typically these systems encapsulate and then transmit medical information to the recipient, without accepting immediate acknowledgment. These methods might be required due to cost constraints. Examples of “store-and-forward” include correspondence via e-mail, fax, or data mule. The main advantage of this form of telemedicine is that no online services are required and the content of the information can be examined as soon as possible [15]. The use of delay tolerance network infrastructure to provide telemedicine in remote areas has also been investigated in [16], and a simple telemedicine system using a digital camera was evaluated in [17]. In that test bed, radiographs on a viewing box were photographed at a remote hospital in South Africa, using a digital camera, and the resultant images were stored in JPEG format and transmitted, later to be read on a PC monitor by radiologists in Durban and Cape Town.

## 3. CoDPON Architecture

CoDPON network, proposed in [5], is a specific data mule system inspired by the air traffic control system. It basically consists of four components: nodes, DACT (data application in transit, data divided into logical units), displacement plans, and the communication protocol. There are three types of nodes: vehicles, peers base stations (PBSs), and hot spots.* Vehicles* (in our case, boats) act as mules, carrying the data spread along the path. The vehicles used on a CoDPON network operate on predefined routes, with prescheduled (days and times) departures and arrivals. All boats have a computer housed in an airtight box and use solid state disks to ensure more robust and persistent storage. The solid state is to prevent damage due to motion of boats.

Peer base stations are fixed nodes, previously mapped, where vehicles make scheduled stops. Peer base stations are usually located on the river shore. Hot spots are special nodes that exist only in areas that have connection to the Internet. They work as a faster gateway between CoDPON nodes. Unfortunately, solutions such as the ones proposed in [1], which reduce latencies equipping the MULEs with an always-on connection (such as a cellular or satellite phone), are not available in some scenarios (e.g., there is a very limited telecommunications coverage in Amazonia). A DACT represents a minimum unit of data transferred between nodes and has a self-meaning that allows pieces of data to be separated from others without losing consistency.

A* displacement plan* is applied to all boats. It contains basic information about the entire route of the boat, including starting point, destination, stopovers, and estimated time of journey and anchoring.* Journey time* is the time required for a boat to travel its entire route.* Anchor time* usually is the time for passengers to embark or disembark at village piers. Each boat has its own displacement plan stored on board, including a table containing the hydrographic distances between peer base stations.

The communication protocol is based on the principle of data mule. It does not require changes in the TCP stack, as is the case with the DTN. Mathematical models that can be referenced to support this project are found in [18].

## 4. Application

**Figure 1:**
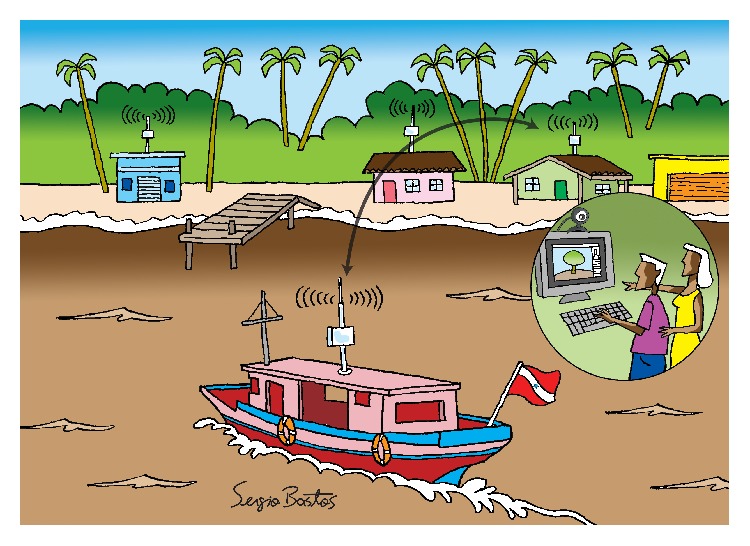
Boats exchanging ultrasound files.

**Figure 2:**
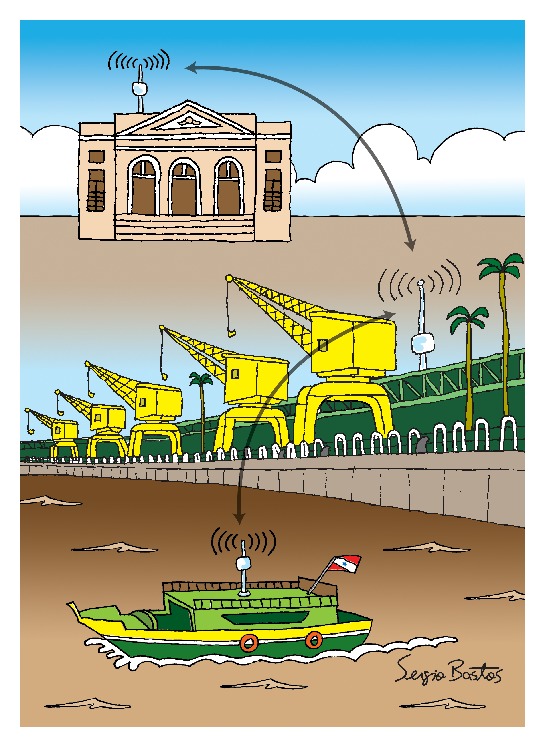
Delivery of ultrasound files at the state capital.

A major difficulty for health care in isolated areas, like the Marajó Archipelago, occurs due to the small number of physicians who reside in the region. The vast majority of those just make periodic visits to the communities in the region. Among the most affected people by the infrequent medical visits in the communities are pregnant women, who need regular monitoring. Hence, we considered the proposed application of remote ultrasound monitoring. The CoDPON system will act as a data mule network, carrying and delivering ultrasound files from the remote communities (Figure 1) to the state capital, Belém, for analysis by physicians (Figure 2).

The ultrasound parameters used in the simulation process were obtained by consulting a team of experts on ultrasounds, including OB∖Gyn doctors in Belém, who operate in the Marajó region. According to the team, the ultrasound examination is a dynamic diagnostic method in which the physicians acquire the ultrasound videos and interpret them in a dynamic way. Two synchronized videos are required for the remote analysis to be efficient: the first video shows the image generated by the equipment and the second video shows the examination process (the position of the transducer being used by health agent on the patient's body).

## 5. Simulation

**Figure 3:**
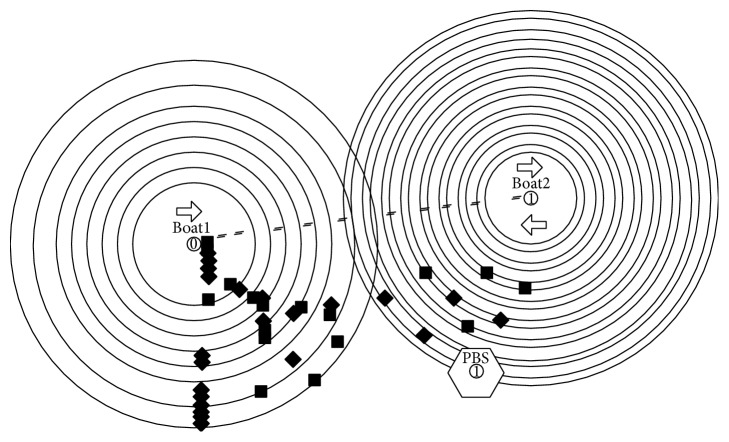
Connection time simulation.

Two open source simulation tools were selected for the evaluation of the scenario described in the Marajó Archipelago. The first software was NS-2, probably the most widely used simulator in the scientific community [19]. The NS-2 wireless module was used to inspect the connection time between boats (in motion) and peer base stations and between a boat and another boat (both in motion) (Figure 3). The other software was the ONE simulator [8], which has rich features available for simulating DTNs with numerous mobility models.

### 5.1. Scenario

**Figure 4:**
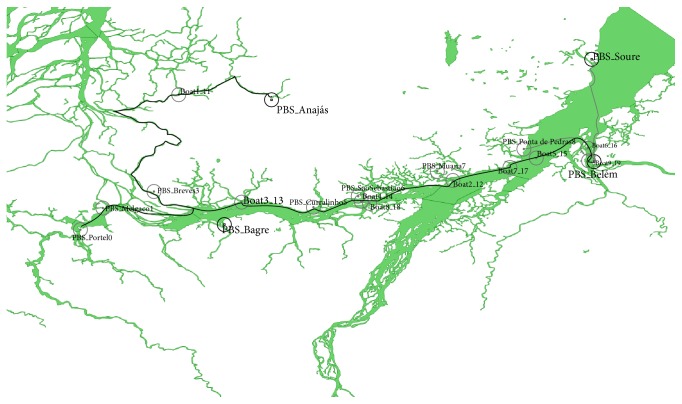
Area overview.

For the simulation, an area of 104,142 Km^2^ (approximately 40,700 sq mi) comprising the Marajó Archipelago, located in the state of Pará, Brazil, was selected (the south part of the Marajo Island is shown in Figure 4). The routes for the boats were created according to local data, obtained from the office of the public boats terminal in the city of Belém (capital of Pará). The boats are equipped with Wi-Fi IEEE 802.11 devices and they follow routes throughout the simulation area, following predefined routes. Each boat has scheduled stopovers at various peer base stations.

### 5.2. Simulation Parameters

For the simulation, various combinations of parameters were selected (Table 1).

The scales of the simulations conducted in NS-2 and the ONE simulator were very different. In the simulation in NS-2, we evaluated the amount of data transmitted between boats and PBSs, which required a finer grain simulation and more specifics about the radio wave communications. The simulations conducted in the ONE simulator concerned the flow of packets over the network, following the end-to-end boat displacement plans.

As some routes are traveled for up to seventeen hours, the time to live (TTL) value was set to 1,122 minutes, 10% more than the value of the maximum travel time. When the TTL expires, the message will be self-destroyed, if it had not been delivered yet.

### 5.3. Traffic Generator

In order to configure the traffic generator for the simulator, the demand of real data loads in Marajó was investigated* in loco*. During the sporadic government health programs, physicians perform one hundred ultrasound exams in one weekend, once a month. In other words, working at maximum capacity, they perform fifty exams per weekend day. If we consider that each ultrasound size, in Mpeg format, is around 5 Megabytes (using the minimum acceptable standards for digital compression of a fetal ultrasound video clip [20]) and we add the video of a health agent performing the examination, 10 Megabytes is a reasonable size for each ultrasound video set. Thus, the traffic generator of the ONE simulator was set up to work at 384 kbps. The value was obtained considering that fifty ultrasounds per day are performed, each generating a 10 MB ultrasound file. Three PBSs, located at the extreme points of the Marajó Archipelago, were selected as sources of video transmissions, all of them sending files to the main PBS in Belém. The average boat speed of 15 Km/h was obtained by GPS* in loco*.

## 6. Results

### 6.1. Simulation in NS2

Once a boat stops at a PBS, it could receive DACTs, due to its large buffer (SSD disk). Therefore, the investigations were concentrated in critical points, that is, when the data transfer occurs while the boats are moving. In the simulation, three different scenarios were tested.

**Figure 5:**
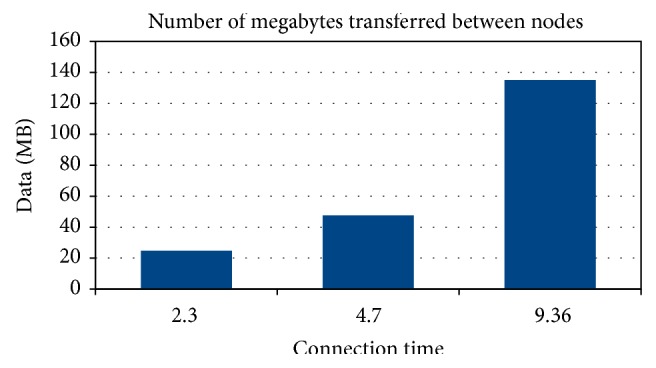
Amount of data transferred.

Scenario 1 shows a boat passing within 50 meters of another boat in the opposite direction. In this case, both boats are in motion. Scenario 2 shows a boat passing within 50 meters of a peer base station, which is a fixed point, without stopping. Scenario 3 shows two boats in movement, moving in the same direction, and one is traveling at twice the speed of the other. In all scenarios, connection times and amount of data transferred were analyzed (Figure 5).

In the first scenario, transmission occurs between seconds 172 and 320 of the simulation time line, which represents a duration of 148 seconds. This results in a total of 2.3 minutes of connection time between the boats. This would allow for the transfer of two ultrasound video sets.

In the second scenario, transmission occurs between seconds 99 and 381 of the time line simulation, which represents a total of 282 seconds. This results in a total of 4.7 minutes of connection time between a boat and a PBS. With these values it would be possible to transfer 47.62 Megabytes, which would allow the transfer of four ultrasound video sets.

**Figure 6:**
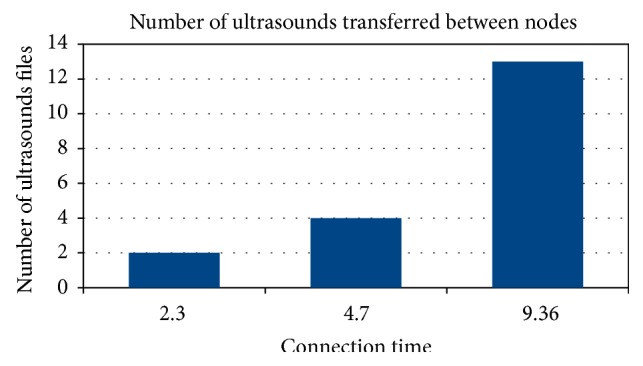
Number of medical exams transmitted.

In the third scenario, transmission occurs between instants 198 and 760 of the time line simulation, which represents a total of 562 seconds. This results in a total of 9.36 minutes of connection time between the boats. With these values, 135.08 Megabytes would be transferred, which would allow the transmission of thirteen ultrasound exams sets (Figure 6).

### 6.2. Simulation in ONE

The map of the Marajó Archipelago was obtained from IDESP (Institute of Economic, Social and Environmental Development of Pará) [21]. The map was post-processed using the open source GIS tool OpenJump, and marked with various locations such as villages, small cities, and the route of the boat lines.

The scenario used in this section aims to transfer ultrasound sets from remote communities of the Amazon to Belém. The ONE simulator scenario comprises 11 PBSs, and only three of them (PBS_Anajás, PBS_Bagre, and PBS_Soure) generated ultrasound exam files. Each boat has an associated route file defined by the OpenJump software, based on real routes obtained from the main boat station in Belém.

Epidemic routing was used in the simulations. The evaluation was performed by changing the number of boats not only between source and destination but also in the entire scenario. The metrics were compared with one boat, three boats, five boats, and seven boats acting in each route.

**Figure 7:**
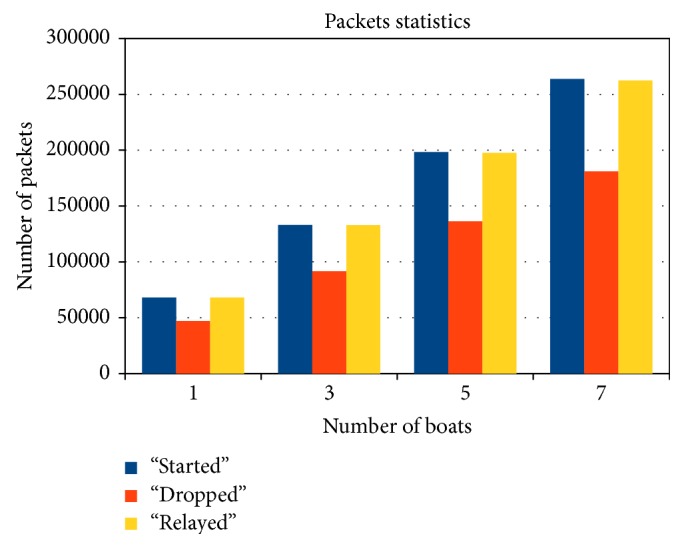
Packet statistics.

Figure 7 shows the relation between started, dropped, relayed, and aborted packets.* Started packets* are the packet transmissions started between network nodes.* Dropped packets* are the packets that are dropped from nodes' buffers and relayed packets are the packets successfully transmitted between nodes. As it can be seen, the number of dropped packets rises with the number of boats, due to the epidemic routing, which indicates a rapid saturation of the network capacity. Basically this is due to having more boats carrying packets the “wrong” way (in the opposite direction of the PBS it should be going to), which end up “wandering” in the network without ever making it to Belém.

**Figure 8:**
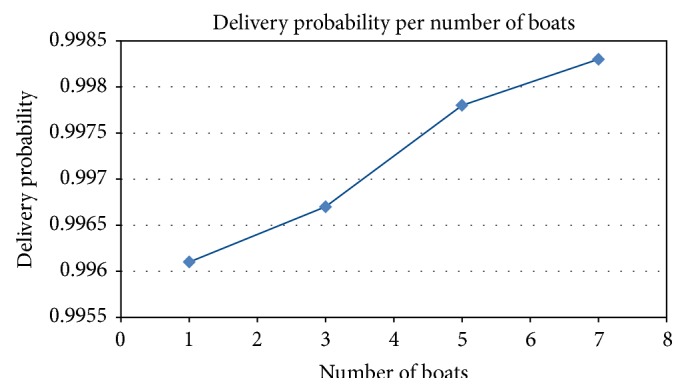
Number of Megabytes.

**Figure 9:**
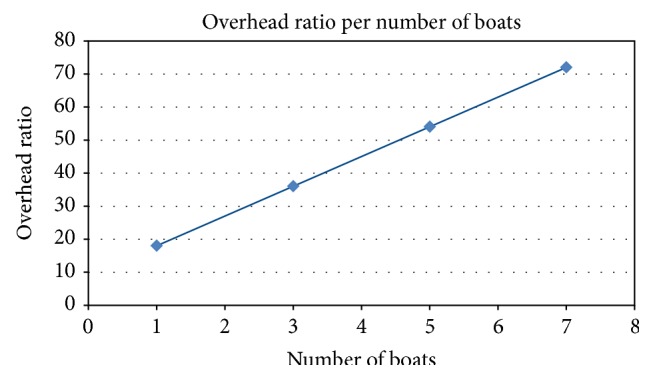
Overhead ratio.

Despite the fact that the delivery probability increases with the increased number of boats (Figure 8), the amount of wandering packets in the network grows in a much larger proportion (Figure 9), also rapidly saturating the network capacity. This is clear when the overhead ratio is observed. For an epidemic-like protocol, the overhead ratio can be interpreted as the number of replicas for each message transmission. In an environment without QoS, this could be a problem because of the large amount of messages to be delivered.

Without a prioritization mechanism, the most important messages can be delivered later and may not be relayed in time. In a CoDPON network, QoS prioritization is based on the Olympic service (QoS architecture that classifies messages in gold, silver, and bronze: gold goes first and then silver, and bronze is considered the best effort) [22] to avoid this problem.

**Figure 10:**
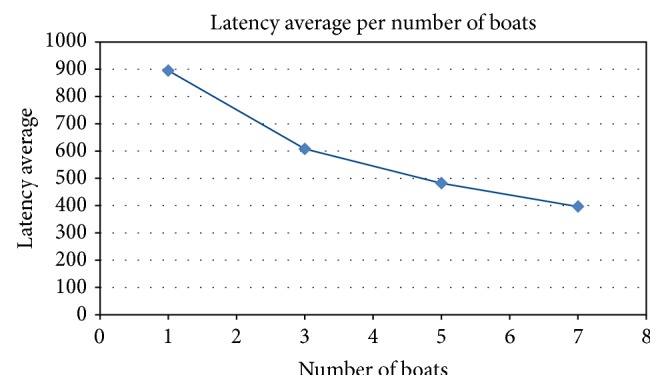
Latency average (in seconds).

Figure 8 shows the delivery probability, which is the probability related to the number of successfully delivered messages. It grows linearly according to the increase in the number of boats. This information can be used by network administrators and local government to estimate how efficient the service is. Depending on the results, more boats can receive the necessary equipment to become nodes in a CoDPON network.  Figure 10 shows the average message delay from creation to delivery. It drops as more boats are used to carry the packets.

The simulation performed can be used to scale the number of boats that will act as data mules in the system and the impact of the use of traditional routing protocols like epidemic routing, for example, to decrease the latency with an increased number of boats.

It is clear that the proposed system has a high delivery ratio with high overhead. But as our goal is to provide medical services to underserviced communities, the delivery of the messages is our priority; thus, the overhead is the issue that needs to be improved but it is acceptable as a starting point.

## 7. Conclusions

The demand for health services is large in the Amazon region. However, due to the lack of communication and transportation infrastructure, many patients who require special care end up unattended. One of the most important aspects of our proposed system refers to the inclusion of health experts in the patients' medical files preliminary analysis. For example, if a large number of neurological problems are detected in the analysis of the remote preliminary examinations files, neurologists may be included in the group of doctors in the boat caravan to the villages.

The simulations in this paper clearly indicate that epidemic routing creates a very large burden on the network, since the packet spreads in all directions without discretion. In a network with known routes, as is the case of CoDPON, where the PBSs may act similar to checkpoint [14] spreading packets, the spread can be controlled to minimize the amount of unnecessary transfers, since there is some control available through the displacement plans.

## 8. Future Work

For future work, we are developing an algorithm that will diffuse messages according to the displacement plan of the boats in a controlled way. That algorithm will be implemented for the ONE simulator and the results will be compared with the existing implementation.

Another topic for further research is to evaluate how to reduce excessive messages sent via the epidemic routing protocol. The Spray and Wait [23] protocol limits the number of copied messages to a predefined maximum and distributes the copies to the contacted nodes until the maximum number of copies has been reached. Supp-Tran [24] optimizes the performance of epidemic routing protocol in terms of delivery probability, overhead ratio, and hop count average. Such approaches and other message distribution protocols will be evaluated to improve our system's performance.

Since components of the proposed system might suffer unpredictable failures, robustness must be introduced. In order to increase efficiency and robustness in the delivery of exam files, we will study the use of fountain codes and UPC codes [25]. According to [26], a fountain codifier is a metaphoric well which produces an infinite quantity of drops of water (packages of codified data). Thus, anyone who wishes to receive a coded file puts a bucket under the well and collects drops until the number of drops in the bucket is sufficient to recover the original file and decode the packages. In the analogy of the CoDPON system, the well would be the checkpoints, and the drops (packages) would be transported by the boats up to the destination (the state capital), which would have a bucket to collect them. If one assumes that the information one wishes to be transferred is a “bucketful of water,” one needs only to generate several drops and feed the bucket (which will be collected at the destination) at the checkpoints, until they are full of original information. The great contribution of fountain codes is that they can retrieve files, even without all the original packages, or it is possible to rebuild a lost package using copies left over from packages already received.

## Figures and Tables

**Table 1:** Simulation parameters.

Parameter	NS-2	The ONE
Flat grid/world size (Km)	2000 × 200	22000 × 20000
Simulation time	1000 sec	216000 (60 hours)
Wireless interface transmit speed (Mbps)	11	11
Wireless interface range (m)	100	100
Radio frequency	2.4 GHz	—
Path loss exponent (rain)	2	—
Average boat speed (Km/h)	15	15
Number of PBS (DTN module in seashore)	1	11
Displacement plans	—	9
